# Assessment of Sexual Dimorphism Through Lateral Cephalogram in Children Aged 10-12 Years

**DOI:** 10.7759/cureus.64107

**Published:** 2024-07-08

**Authors:** Prakriti Sagarika, Sujitha Ponraj, Kavitha Ramar, Rajakumar S

**Affiliations:** 1 Pediatric and Preventive Dentistry, SRM Kattankulathur Dental College and Hospital, Chennai, IND

**Keywords:** dental xray, forensic, children, lateral cephalogram, sexual dimorphism

## Abstract

Introduction: Forensic dentistry integrates interdisciplinary scientific knowledge to produce accurate and reliable forensic statements. Anthropometry, essential since its introduction by Alphonse Bertillon in 1882, describes human body shapes and has significant forensic applications. This study focuses on sexual dimorphism, phenotypic differences between males and females, using lateral cephalometric measurements to determine sex in children aged 10-12 years from the Chengalpattu population.

Materials and methods: The cross-sectional study included 80 participants (40 boys and 40 girls). Lateral cephalograms were analyzed using Ceph Ninja Pro software to obtain 15 cephalometric measurements. Statistical analysis using SPSS Software (Version 22.0) involved t-tests to identify significant differences (P<0.05). Discriminant function analysis assessed the predictive power of these variables.

Results: Seven variables showed significant differences between sexes. Discriminant models based on these variables determined sex with varying reliability. Ramus length was the most reliable predictor (81%), while maxillary length had the lowest reliability (62%).

Discussion: The study’s findings align with existing literature, indicating the robustness of ramus length for sex determination. However, the low reliability of maxillary length contrasts with studies that found it useful for sex differentiation, suggesting variability across populations and age groups. Combining multiple cephalometric variables improved accuracy, consistent with previous research.

Conclusion: Lateral cephalograms are effective for assessing sexual dimorphism in children. The study supports the forensic and clinical utility of cephalometric measurements and calls for further research with diverse populations and advanced imaging techniques to enhance method reliability and applicability.

## Introduction

Forensic dentistry encompasses an interdisciplinary approach that applies verified scientific knowledge to formulate precise and reliable statements [1]. Anthropometry, a method for describing the human body's shape, has been integral to forensic science and dentistry since 1882, when Alphonse Bertillon, a French police expert, introduced a system of criminal identification based on anthropometric measurements [1]. Anthropometric characteristics, which include sex, shape, and overall form, are closely interconnected and significantly influence the individual's internal structure and tissue composition, which are in turn affected by both environmental and genetic factors [1,2]. The utilization of anthropometry can arise in various contexts, including natural occurrences such as war casualties, air crashes, road accidents, and disasters like earthquakes, floods, and fires, as well as intentional acts such as deliberate mutilation, disfigurement, or other forms of postmortem trauma inflicted on the deceased body [3,4].

Sexual dimorphism, the phenotypic differences between males and females of the same species, is a fundamental aspect of human biology [5]. These differences extend to craniofacial structures, making them a focus of interest in various fields such as anthropology, forensic science, pediatric dentistry, and orthodontics [5]. The lateral cephalogram, a two-dimensional radiographic image of the head and face in profile, has long been utilized to assess craniofacial morphology. Its potential in elucidating sexual dimorphism has garnered attention due to its non-invasive nature and widespread availability in clinical and research settings [6].

Numerous studies have explored sexual dimorphism using lateral cephalograms, investigating differences in skeletal dimensions, proportions, and morphological features between males and females. These investigations often encompass various craniofacial regions, including the cranial base, maxilla, mandible, and soft tissue profiles [7]. Understanding the extent and nature of sexual dimorphism in these structures holds implications for a range of applications, from forensic identification to orthodontic treatment planning [7].

Understanding the extent and nature of craniofacial dimorphism between sexes not only contributes to our knowledge of human biological variation but also has practical implications in fields such as forensic anthropology, facial reconstruction, and orthodontic diagnosis and treatment planning. This study addresses the need for reliable methods in forensic investigations, aiding in the determination of sex when traditional identification techniques are insufficient. Additionally, the findings have clinical applications in pediatric dentistry, orthodontics, and maxillofacial surgery, guiding treatment plans tailored to gender-specific craniofacial morphology. Hence, the aim of this study is to assess the reliability of parameters derived from lateral cephalograms to identify the sex of an individual in the Chengalpattu population.

Furthermore, insights into sexual dimorphism contribute to our understanding of human biological variation and evolutionary processes. Ultimately, this research informs forensic anthropology practices and may influence policy-making in forensic science and healthcare.

## Materials and methods

The study was approved by the institutional ethics committee prior to its start (SRMIEC-STO533-754). This cross-sectional study utilized pre-treatment lateral cephalograms from 80 participants who visited our hospital for orthodontic treatment, consisting of an equal number of boys and girls (40 each) aged between 10 and 12 years. The sample size was calculated using G Power Software with a power of 0.80. Inclusion criteria involved individuals with no history of facial trauma, orthodontic treatment, or cosmetic surgery. Exclusion criteria encompassed individuals with a history of orthodontic treatment or surgery, maxillofacial trauma, and those with low-quality X-rays.

The study involved acquiring 15 cephalometric measurements, which included both linear and angular assessments: anterior cranial base: measurement from the sella to the nasion. Posterior cranial base: measurement from the sella to the basion. Jarabak's sum (saddle + articulare + gonial angle): sum of the saddle angle, articulare angle, and gonial angle. Upper gonial angle: the angle formed by the lines tangent to the posterior and inferior borders of the ramus. Lower gonial angle: the angle between the ramus plane and the mandibular plane. Total effective maxillary length: distance from the condylion to point A. Ramal length: the vertical distance from the condylion to the gonion. Chin prominence: the horizontal distance from pogonion to nasion perpendicular. Mandibular body length: distance from the gonion to the menton. Facial angle: the angle formed by the facial plane and the Frankfort horizontal plane. Mandibular plane angle: the angle formed by the mandibular plane and the Frankfort horizontal plane. Facial height index: the ratio of lower facial height to total facial height. Facial height ratio: the ratio of upper facial height to lower facial height. Mandibular length: distance from condylion to gnathion. Maxillary length: distance from point A to point B.

**Figure 1:**
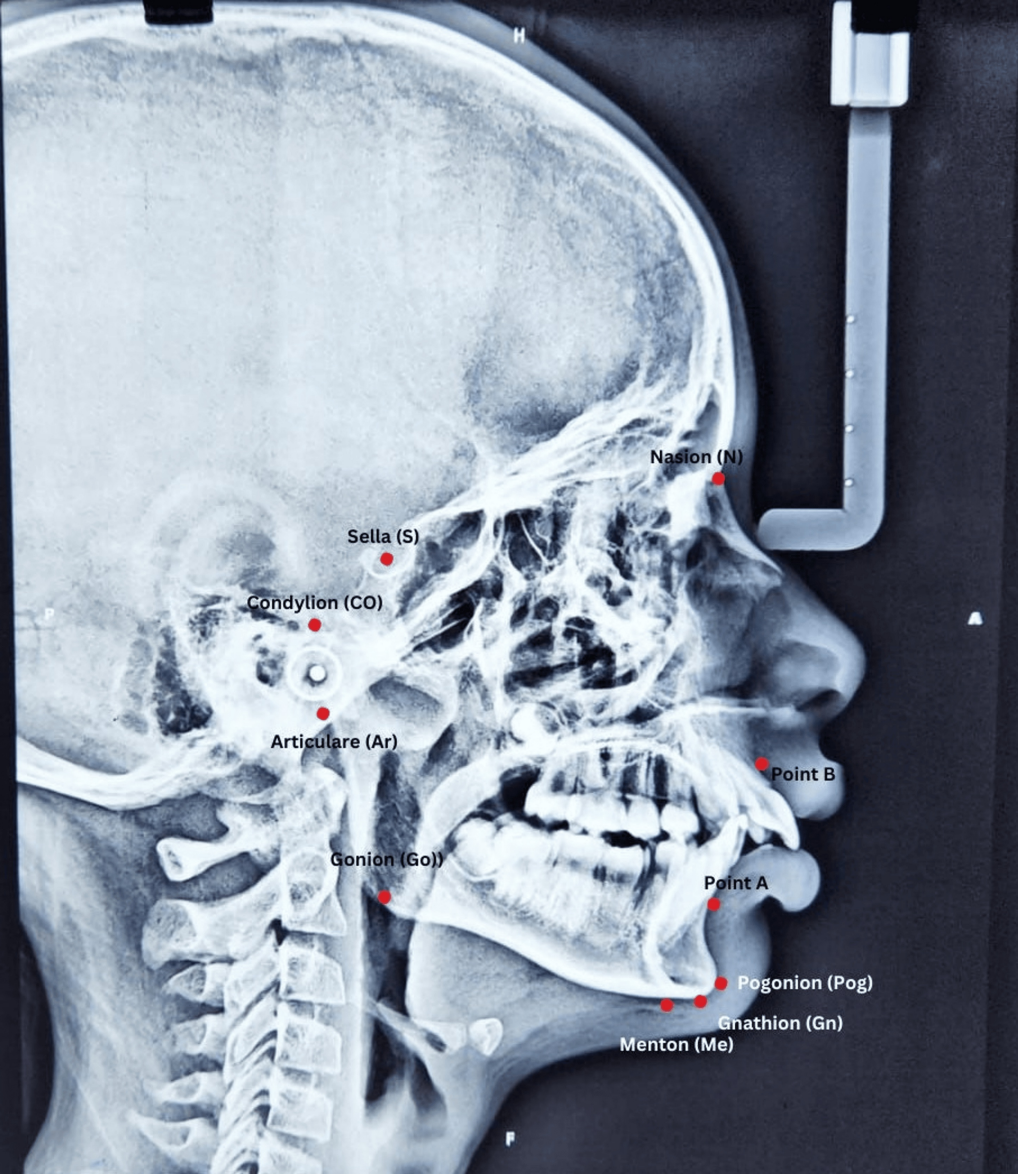
Lateral cephalogram of the female patient aged 10 years participated in this study with landmarks of the parameters marked

Anatomical points on the lateral cephalogram were identified and outlined with the assistance of Ceph Ninja Pro software. This software automatically calculates values for both linear and angular variables, reducing the risk of human errors. Figure 1 shows an example lateral cephalogram with all the points marked for the measurement.

The variables obtained from cephalometric analysis were subjected to a statistical analysis conducted by SPSS Software (Version 22.0). Descriptive statistics were obtained in the form of mean and standard deviation. All variables underwent significance testing using a t-test. A significance level of P<0.05 was set for statistical significance.

Discriminant functional analysis (DFA) was performed to evaluate the accuracy of sex determination using various craniofacial measurements. The analysis included calculating Wilks’ lambda to assess each variable's discriminative power, F-values to test statistical significance, and the predictability percentage to determine the correct classification rate. The collective predictability of the model was 96%, indicating a high accuracy in sex determination.

## Results

Among the 16 variables observed, seven parameters showed statistically significant differences between males and females. Subsequently, these seven variables underwent discriminant function analysis to assess how effectively each variable could predict the gender of the individual under consideration (Table 1).

**Table 1:** Comparison of lateral cephalogram parameters between male and female participants T-test has been performed for comparison between males and females. *p<0.05 indicates statistical significance

Parameters	Gender	Mean	Std. Deviation	P-Value
Anterior Cranial Base	Female	45.8500 mm	2.08051 mm	.002*
	Male	48.0703 mm	3.83431 mm	
Posterior Cranial Base	Female	23.8685 mm	2.07292 mm	.144
	Male	24.7748 mm	3.28213 mm	
Jarabak's Sum (Saddle+Articulare+Gonial Angle)	Female	391.4442 mm	4.70658 mm	.004*
	Male	397.6027 mm	5.37526 mm	
Upper Gonial Angle	Female	55.2445°	4.39572°	.793
	Male	54.9913°	4.22462°	
Lower Gonial Angle	Female	72.3867°	3.89791°	.333
	Male	73.3465°	4.86671°	
Total Effective Maxillary Length	Female	33.4438 mm	1.87215 mm	.159
	Male	34.3263 mm	3.44840 mm	
Ramal Length	Female	26.5758 mm	3.38955 mm	.0057*
	Male	29.8965 mm	2.64606 mm	
Chin Prominence	Female	3.4080 mm	2.00746 mm	.957
	Male	3.3874 mm	1.30936 mm	
Mandibular Body Length	Female	48.53525 mm	2.993107 mm	.942
	Male	48.59400 mm	4.164498 mm	
Facial Angle	Female	80.7427°	3.92176°	.0034*
	Male	84.1395°	3.81807°	
Mandibular Plane Angle	Female	31.3475°	4.96193°	.835
	Male	31.5805°	4.98656°	
Facial Height Index	Female	.6625	.07351	.002*
	Male	.7255	.18253	
Facial Height Ratio	Female	56.8675	1.78977	.0032*
	Male	59.7705	2.16735	
Mandibular Length	Female	65.1515 mm	9.53708 mm	.122
	Male	68.3215 mm	8.55107 mm	
Maxillary Length	Female	54.2298 mm	4.26019 mm	.0045*
	Male	58.2450 mm	7.39152 mm	

For every significant variable, a discriminant model was established, featuring distinct formulas for males and females. By inputting numerical values into the respective formulas, the individual's gender was determined based on which formula yielded a higher result. The accuracy of these variables was then assessed to gauge their predictability.

Individually, the highest reliability was with Ramus length in determining sex, with a consistency of 81%. Conversely, Maxillary length displayed the lowest reliability at 62%. On average, the remaining variables demonstrated a reliability percentage above 60% (Table 2).

**Table 2:** Predictability of parameters DFA was performed by calculating Wilks’ lambda to assess each variable's discriminative power and F-values to test statistical significance. F, F statistic; DFA, discriminant functional analysis

Variable	Wilks’ Lambda	F	Predictability (%)
Anterior Cranial Base	0.861	5.562	76
Jarabak's Sum (Saddle+Articulare+Gonial Angle)	0.814	5.323	75
Ramal Length	0.812	11.352	81
Facial Angle	0.856	6.426	65
Facial Height Index	0.916	4.392	70
Facial Height Ratio	0.902	7.625	69
Maxillary Length	0.890	8.104	62

## Discussion

The present study aimed to evaluate sexual dimorphism in children aged 10-12 years using lateral cephalometric measurements. Among the 16 variables initially observed, seven demonstrated statistically significant differences between males and females. These variables were subjected to discriminant function analysis to determine their efficacy in predicting gender.

Our finding that ramus length is the most reliable predictor of gender, with an accuracy of 81%, aligns with several other studies. For instance, Franklin et al. (2008) reported that mandibular ramus height was a significant indicator of sexual dimorphism in their study on a South African population, demonstrating similar high reliability in gender determination [8]. Similarly, studies by Kharoshah et al. (2010) confirmed that the ramus was a significant variable for sex differentiation in an Egyptian sample, emphasizing its robustness across different populations [9].

Conversely, our study found the maxillary length to have the lowest reliability at 62%. This finding contrasts with some studies, which have identified the maxilla as a reasonably good predictor of sex. Patil and Mody (2005) reported that maxillary measurements were effective in distinguishing gender in a sample of Indian children, though their sample included a wider age range, which might account for the differences observed [10]. Additionally, Hsiao et al. (2010) observed that maxillary dimensions were useful in sex differentiation in Taiwanese children but noted variability based on age and developmental stage [11]. This suggests that maxillary length may vary significantly with age and population, impacting its utility as a reliable marker for sexual dimorphism in specific age groups.

The combined use of multiple cephalometric variables yielded higher accuracy rates for gender determination, a finding consistent with previous research. Giles and Elliot (1962) demonstrated that using multiple discriminant function variables significantly enhances the accuracy of sex estimation compared to single variable analyses [12]. Similarly, Franklin et al. (2007) highlighted that a multivariate approach, incorporating several cephalometric measurements, provided a comprehensive assessment with improved accuracy [13].

Further support for the importance of multiple variables in sex differentiation comes from studies on craniofacial growth patterns. Nanda (1983) emphasized that growth patterns of the craniofacial complex differ between males and females, particularly during the pubertal growth spurt, making it crucial to consider multiple measurements for accurate assessment [14]. Additionally, Behrents (1985) noted significant differences in mandibular growth trajectories between genders, reinforcing the relevance of mandibular variables in sex determination [15].

The development of discriminant models for males and females in our study provides a practical tool for gender determination in forensic and clinical settings. These findings are supported by the work of Steyn and Iscan (1998), who emphasized the application of discriminant function analysis in forensic anthropology for accurate sex determination using skeletal remains, including craniofacial measurements [16]. Moreover, Rai et al. (2007) demonstrated the applicability of cephalometric analysis in forensic odontology for identifying gender in an Indian population, further validating our approach [17].

While our study demonstrates significant findings, it is important to consider its limitations. The sample size, although adequate, could be expanded to include more diverse populations to enhance the generalizability of the results. Additionally, longitudinal studies tracking the same cohort over time would provide insights into how these cephalometric variables evolve with age and how early these differences manifest.

Further research should also explore the integration of advanced imaging techniques and 3D cephalometry to potentially increase the accuracy and reliability of gender determination methods. Comparing results across different populations and age groups would further validate the robustness of the identified variables.

## Conclusions

In conclusion, this study supports the use of lateral cephalograms for assessing sexual dimorphism in children aged 10-12 years. Ramus length emerged as the most reliable predictor while combining multiple cephalometric variables further improved gender determination accuracy. These findings align with existing literature, underscoring the potential of cephalometric measurements in forensic and clinical applications. Future research should aim to broaden the scope and scale of studies to enhance the applicability and accuracy of these methods in diverse populations.

## References

[1] Iscan MY (2005). Forensic anthropology of sex and body size. Forensic Sci Int.

[2] Krishan K (2006). Anthropometry in forensic medicine and forensic science anthropometry. J Forensic Sci.

[3] Kosa F (2000). Application and role of anthropological research in the practice of forensic medicine. Acta Biol Szeged.

[4] Krogman WM (1955). The human skeleton in forensic medicine. Postgrad Med.

[5] Missier MS, Samuel SG, George AM (2018). Facial indices in lateral cephalogram for sex prediction in Chennai population - a semi-novel study. J Forensic Dent Sci.

[6] Qaq R, Mânica S, Revie G (2019). Sex estimation using lateral cephalograms: a statistical analysis. Forensic Science International: Reports.

[7] Ningtyas AH, Widyaningrum R, Shantiningsih RR, Yanuaryska RD (2023). Sex estimation using angular measurements of nasion, sella, and glabella on lateral cephalogram among Indonesian adults in Yogyakarta. Egypt J Forensic Sci.

[8] Franklin D, O'Higgins P, Oxnard CE, Dadour I (2008). Discriminant function sexing of the mandible of indigenous South Africans. Forensic Sci Int.

[9] Franklin D, Oxnard CE, O'Higgins P, Dadour I (2007). Sexual dimorphism in the subadult mandible: quantification using geometric morphometrics. J Forensic Sci.

[10] Kharoshah MA, Almadani O, Ghaleb SS, Zaki MK, Fattah YA (2010). Sexual dimorphism of the mandible in a modern Egyptian population. J Forensic Leg Med.

[11] Patil KR, Mody RN (2005). Determination of sex by discriminant function analysis and stature by regression analysis: a lateral cephalometric study. Forensic Sci Int.

[12] Hsiao TH, Tsai SM, Chou ST, Pan JY, Tseng YC, Chang HP, Chen HS (2010). Sex determination using discriminant function analysis in children and adolescents: a lateral cephalometric study. Int J Legal Med.

[13] Giles E, Elliot O (1963). Sex determination by discriminant function analysis of crania. Am J Phys Anthropol.

[14] Nanda RS (1983). Growth patterns in the transition from mixed to permanent dentition. J Clin Orthod.

[15] Behrents RG (1985). Growth in the aging craniofacial skeleton. https://catalog.hathitrust.org/Record/000409370.

[16] Steyn M, Iscan MY (1998). Sexual dimorphism in the crania and mandibles of South African whites. Forensic Sci Int.

[17] Rai B, Kaur J, Anand SC, Dhattarwal SK (2007). Mandibular and maxillary fragments: identification using cephalometry. J Forensic Dent Sci.

